# Society Proceedings

**Published:** 1902-06

**Authors:** 


### SOCIETY PROCEEDINGS.

### PENNSYLVANIA STATE VETERINARY MEDICAL ASSOCIATION.

THE annual meeting was opened at Odd Fellows' Temple, Philadelphia, Pa., on March 4, 1902. The meeting was called to order by the President, Dr. Harger, at 10.30 A. M. The following members were present: Drs. W. G. Benner, Doylestown; John L. Bradley, Mercersburg; J. F. Butterfield, South Montrose; M. J. Chrisman, Sugar Grove; M. J. Collins, Myerstown; Jacob Helmer, Scranton; J. C. Kingsland, Canton; James R. Mahaffey, Wilmington, Del.; S. W. Mathews, Concordville, Pa.; J. C. McNeil, Pittsburg; J. C. Michener, Colmar; Otto Noack, Reading; J. H. Oyler, Harrisburg; John B. Raynor, Milestown; Thomas B. Rayner, Chestnut Hill; W. L. Rhoads, Lansdowne; W. H. Ridge, Trevose; J. T. Ross, Frankford; J. W. Sallade, Auburn; A. W. Weir, Greenville.

The Philadelphia members present were J. M. Carter, H. B. Cox, H. B. Felton, J. T. Ferley, S. J. J. Harger, W. Horace Hoskins, J. D. Houldsworth, C. J. Marshall, James T. MacAnulty, Leonard Pearson, E. M. Ranck.

The visitors present were Drs. L. D. Horner, Woodstown, N. J.; F. H. Schneider, Ninth and Tioga Streets, Philadelphia; Charles Lenhart, Dover; S. H. Johnson, W. D. Martin, and George Fuller, of Philadelphia; also Messrs. F. H. Bradley, A. H. Cheney, G. A. Dicks, G. V. Foster, W. D. Fuller, G. H. Hart, J. H. Morse, and M. H. White, Jr., students at the Veterinary Department of the University of Pennsylvania; also Messrs. George Snyder and George Teufel, Philadelphia, Pa.

The minutes of the previous meeting were read and approved. President Harger delivered his annual address, after which the following officers were elected for the ensuing year:

President, W. L. Rhoads, Lansdowne, Pa.; First Vice-President, J. F. Butterfield, South Montrose; Second Vice-President, A. W. Weir, Greenville; Third Vice-President, W. G. Benner, Doylestown, Pa.; Treasurer, Francis Bridge, No. 228 North Fifty-third Street, Philadelpha; Recording Secretary, C. J. Marshall, No. 2004 Pine Street, Philadelphia; Corresponding Secretary, E. M. Ranck, No. 422 N. Forty-first Street, Philadelphia; Board of Trustees—Leonard Pearson, Thirty-sixth and Spruce Streets, Philadelphia; W. H. Hoskins, No. 3452 Ludlow Street, Philadelphia; Thomas B. Rayner, Chestnut Hill, Philadelphia; W. H. Ridge, Trevose; N. Rectenwald, No. 89 Washington Avenue, Pittsburg.

A recess was then given for the collection of dues. After adjournment a report of the Board of Trustees was called for. Dr. Pearson requested that his report be postponed. Dr. Harger requested leave of absence for an hour, and Dr. J. C. Michener took the President's chair during his absence. Reports of County Secretaries were then read by the Corresponding Secretary, Dr. Ranck. A report of the delegates to the American Veterinary Medical Association meeting in September last was given by Dr. Hoskins. Drs. Hoskins and Noack urged dropping of the clinics at the American Veterinary Medical Association meetings. Veterinary

Medical Association of New Jersey: Dr. Pearson made a few appropriate remarks. New York State Veterinary Medical Society: Dr. Carter was called. He did not attend the meeting, but reported that the late Dr. R. S. Huidekoper had represented the Pennsylvania veterinarians at the meeting at Ithaca. Dr. Ranck spoke of the Dr. Morris affair at Atlantic City and what had been done by the American Veterinary Medical Association, and also what had not been done by the New York State Association, and asked Dr. Hoskins for an explanation. Dr. Hoskins reported that Dr. Huidekoper attended this meeting, and said nothing definite was done. The Association dillydallied with the subject, and the main effort seemed to be to avoid the issue, and the question was laid over for one year. He feels the Association should be censured for its cowardice, and will introduce a resolution later to this effect. Keystone Veterinary Medical Association: Dr. Ridge and Dr. Marshall spoke of the good work this Association was doing, and urged more frequent attendance of the local Association by the local men. Schuylkill Valley Veterinary Medical Association: Dr. Schneider was called and gave the following report: "This Association was formed six years ago. It now has thirty active members, and meets quarterly. The last meeting was held last December. Some very good papers were read." Dr. Pearson thought that many other veterinarians would attend these meetings if they were invited. He suggested that the JOURNAL would be glad to print notice if received in time. Dr. Noack said that they used to send invitations broadly; but as few accepted them they had discontinued it. Dr. Ranck said that a secretary is not excusable if every veterinarian, every newspaper and journal of note does not receive notice, even if they do not attend or send representatives. He thought we should be generous with our invitations. Dr. Noack thought it an unnecessary expense to send invitations to those who would not attend. Dr. Ranck suggested that the proceedings should be printed. Dr. Noack said that reports were sent to Dr. Hoskins and not answered. Dr. Hoskins thought not, and was defended by Dr. Ridge, who said that Dr. Hoskins always answers letters promptly.

The Treasurer's report of the Pennsylvania State Veterinary Medical Association was read by Dr. Bridge.

Dr. Hoskins thought that the condition of our treasury is open to improvement. He thought the money should be used for the benefit of our Association, and urged that it should be distributed for the benefit of our profession throughout the State in a better enforcement of the State veterinary laws and in creating a stronger public sentiment for veterinary representation on all Boards of Health.

### Reports of Committees.

Dr. Ranck read a report from Dr. Sallade, of the Committee on Legislation. He suggested that all veterinarians should register every three to five years. Dr. Noack said that a bill was introduced by the Schuylkill Valley Veterinary Medical Association; that the laws of 1889 were faulty. The new bill was to amend the old one of 1889, whereby all veterinarians would be required to register again. He read the proposed amendment. Dr. Hoskins read a report of this committee, and urged the importance of re-registration. By a re-registration money might be obtained for carrying on the work of prosecuting illegal practitioners. Three prothonotaries have been called before the courts to show that certain unwarranted registrations had been made by them. Two men are now serving time and two others are being prosecuted. He hoped that Dr. Noack's paper would be presented to our Committee on Legislation, and that some action would be taken by this Association. Letters are being sent to the prothonotaries of different counties to have the lists of registration verified. Dr. Pearson mentioned the act passed by the last Legislature to provide for the milk-supply of cities of the second class. He thought the trend of this bill was good, but a little too bold. This law has not been put into effect by a single city. The authorities are afraid to put it into operation. It was drawn up by physicians of Pittsburg who lack practical experience. This shows the need of proposed legislation being thoroughly considered by men of practical experience prior to being offered to legislative bodies.

### Committee on Intelligence and Education.

No members of this committee being present, the work was laid over until later. Dr. Pearson thought it was too important to pass over this subject, and offered a verbal report. He described the changes being made at the Veterinary Department of the University of Pennsylvania. The reasons for the changes were twofold: First, the growth of the University of Pennsylvania was so great that our school was being hidden; second, the medical department wanted a somewhat quiet and retired place for a $600,000 laboratory building. The architects are now working on the plans for our new building, which will be a three-story building, where plenty of room will be obtained, and one suitable room for the use of our Association. The plans are for the best-equipped school in any English-speaking country.

### Committee on Sanitary Science and Police.

Passed over for the present.

### Committee on Animal Husbandry.

Dr. Ridge was called as a member of this committee, but made no remarks. Dr. Pearson spoke of the bill before Congress for the purpose of certifying to the qualifications of stallions suitable to get cavalry horses, this committee to be composed of practical horsemen. The general purpose of the bill is to be similar to the Imperial Horsebreeders' Association of Europe. The trend of the bill will be useful if it becomes a law. Our horses are, generally speaking, becoming nondescripts, and we should breed for some specific type. The object of this bill is to encourage the breeding of a class of horses suitable for cavalry uses. Older countries have found this plan necessary. Similar commissions are bringing them good results. A weak point of the bill as it now stands is that it is composed of six officers and twelve practical horsemen, but no veterinarians. We feel that our profession should be represented. As the veterinarian's training and experience are essential factors and a safeguard to this line of work, we should spare no effort in making this fact patent to those concerned before it is too late. The matter is before a committee in Congress. Wadsworth, of New York, is chairman of the committee. There is one member from Pennsylvania on this committee. Dr. Ridge moved that the subject be referred to the Committee on Resolutions. Dr. Rhoads offered an amendment to have the subject widened, if possible, to make it more general. This ended the afternoon session.

After the banquet the President called the meeting to order at 8 P. M. Dr. Marshall made a report for the Committee on Sanitary Science and Police, which was adopted as read. Dr. J. C. Michener made a report for the Committee on Animal Industry, which was filed with the Secretary. Dr. Thomas B. Rayner discussed the paper, and cited a case where a whole herd was tested with tuberculin, and every animal reacted and was sold clandestinely in Bucks County. As the former owner was a millionaire, he did not see that he was excusable for this unappreciated philanthropy. Dr. Pearson spoke of the difficulty of estimating the value of animals in the United States, and explained why there was so much difference in the reports given out by the Bureau of Animal Industry and the one published by Sequard Powers, of Chicago, and the Census Bureau. This subject was discussed by several members, and it was decided that the *American Agriculturist* gave the most satisfactory statistics. He thought there was a lack of horses in the United States. There did not seem to be enough to meet the demand. The report from the Bureau of Animal Industry for 1900 has been challenged, because of the difference of opinion between the Bureau of Animal Industry and the Superintendent of the Census. Dr. McNeil, of Pittsburg, in speaking of the average price of working horses said that he did not believe they could be bought for $55 a head. They are not as cheap as formerly. He thought there was an advance of $20 or $30 a head on carload lots more than last year. Dr. Hoskins explained why the average price was so low. In making up the average price of horses, bronchos and cheap Texan horses are figured in this average. He thought there was no doubt but what the price of good horses is higher than it had been for years. He preferred to believe the statement made by the Census Bureau to that made by the Department of Agriculture. The vast numbers of horses sent to South Africa has taken out the cheaper class. Dr. McNeil said that the Texan pony is nearly extinct. The same is true of the native cattle of Texas, as Texas cattle are mixed with Northern cattle and are becoming larger and more suitable for beef purposes. Dr. Rhoads moved that this report be accepted as read, which met with the approval of the Association.

The next subject was the admission of new members. There was but one candidate. Dr. Hoskins, as a member of the Board of Trustees, thought that one application is a small quota for the Board, and that we should make greater efforts to bring in new members. Dr. Felton agreed with Dr. Hoskins. George S. Fuller, of Philadelphia, was elected as a member of the Association by acclamation, and was introduced by President Harger, and made a few appropriate remarks.

The next in order was new business. Dr. Ranck reported and read a letter from Dr. Bittles, who paid dues to March, 1902, and requested that his name be dropped from the list. Dr. Pearson moved that the letter be reported to the Board of Trustees. Dr. Rhoads spoke of the vast amount of work which devolves on the Corresponding Secretary, and proposed that

$50 be appropriated to the Corresponding Secretary for his work. Dr. Hoskins spoke in favor of the motion. He thought that a certain sum of money should be set aside for the use of the Corresponding Secretary. The motion was adopted.

Dr. Rhoads advocated the creation of a Press Committee of seven members, and offered a motion to that effect. He thought that they should be appointed by the President, so that they can be chosen from the sections of the State where the meeting is to be held. Dr. Noack thought that cartoons and ridiculous-appearing pictures should not accompany the reports of our Association, as has been done in times past. Dr. Hoskins thought that Dr. Noack's objections are one reason for having a Press Committee, and that with such a committee we could put such subjects as we wish before the public.

Dr. Hoskins thought that our State meat-inspection is the greatest farce of modern times, and believed that under the present system we should recommend Western dressed meats in preference to our city dressed meats, and suggested this subject for the consideration of the Press Committee. This is a subject to which ordinary newspaper correspondence cannot do justice. Dr. Williams agreed with the other speakers, but thought that it was difficult to get any satisfaction from the present administration or from the newspapers.

Dr. Ranck then read an application from Dr. H. B. Cox, of Philadelphia, who was elected to membership.

In reference to meat-inspection, Dr. Pearson thought that influence may defeat any recommendation we may make, because the city officials fear that a large sum of money would be necessary to carry on this work. If a tuberculous case is condemned it is for the use of the public, and the public should help to bear some of the loss. Would it not be better to establish a public fund and partly remunerate the loss? It might come from the State or counties or cities. Under the present appropriation it is not possible for the State to pay for all tuberculous animals. If this could be done Dr. Pearson thought the butcher would hail the meat-inspection service, because then the condemned animal would not be an entire loss to him, as it is at the present time. Dr. McNeil asked how much it would cost the State to pay for the cases during the past year. Dr. Pearson said that it could not be answered accurately, as it would not be necessary to have all tuberculous meat condemned. Dr. Hoskins said under the present system of meat-inspection that after 2 P. M. the Federal inspection is declared off, and there was no inspection for the balance of the day. The suspicious cases are kept by the butchers and dressed during the time of no inspection. The greatest opposition he encountered in his late mayoralty campaign was because of his opinion of the city meat-inspection. Dr. Michener recommended that Dr. Pearson's plans be advocated by utilizing in some way the reacting animals. Local butchers are often caught with tuberculous cattle, and these losses are dropped back to the farmer. Some provision should be made to overcome this trouble, but the only remedy is an appropriation of more money to carry on this work.

Dr. McNeil recommended that a committee of five be appointed for the purpose of devising a better system of meat and milk-inspection. Dr. Ranck suggested that seven would be a better number. This was accepted by Dr. McNeil. He thought it would cost Philadelphia $400,000 a year to carry on a proper meat- and milk-inspection. Dr. Ranck asked if the work carried on by Dr. McNeil's proposed committee would not properly fall to our new Press Committee? Dr. Rhoads seconded the motion, which was adopted.

### Unfinished Business.

Dr. Rhoads asked for an explanation of what had been done with the money appropriated by the Association last year. Dr. Hoskins said, in answer, that the $200 appropriated last year has not yet been touched, owing to the unfair statements made by certain members at that time. The meeting was adjourned at 9.30 P. M., to meet at 10 A. M. the next day. Before adjournment Dr. Rhoads spoke about the remarks made by him last year in reference to this appropriation. He still believes, as he did at that time, that it is proper to appropriate the money to be used by the Examining Board, but objected to the way in which the motion was carried out.

### Wednesday, March 5, 1902.

The meeting was called to order by President Harger at 10.30 A. M. Among the letters read by Dr. Ranck was a request to be dropped from membership. It was referred to the Board of Censors. Dr. Jobson read a report from the Committee on Animal Husbandry. The report was filed with the Secretary. A report from Dauphin County by Clinton F. Keiter, and one from Venango County by S. J. Swift were read. Dr. Jacob Helmer read a very carefully prepared report as Chairman of the Committee on Intelligence and Education. This is one of the most thorough and complete reports ever made by this committee. Dr. Raynor made a motion, which was unanimously adopted, that a vote of thanks be extended to Dr. Helmer for his excellent report. Dr. Hoskins moved that a permanent Committee on Membership be appointed, which was carried out. Dr. Felton, in discussing Dr. Helmer's report, wanted to bring special attention to the report gotten out by Brimhall and Wilson, of Minnesota, on the subject of "Hemorrhagic Septicæmia." He also spoke in favor of having a permanent Committee on Membership.

Dr. Remington, a representative of E. R. Squibb & Son, of New York, wished to explain to the Association the advantages of using acetic-acid preparations. Some of the members objected, on the ground that it would be establishing a precedent to allow instrument makers and drug firms to present subjects of this kind to the Association. After a thorough discussion of the subject by the members it was decided that it was for the advantage of our members to have an opportunity to learn of the best forms of drugs and the most useful instruments; consequently Dr. Remington was given the floor, and described fully the advantages to be derived from acetic-acid preparations; that they would be cheaper for our use and fully as reliable as the alcoholic extracts that are universally used at present. Dr. Helmer, of Scranton, asked Dr. Remington the prices of some of the acetic-acid preparations, and claimed that he had not found them to be any cheaper than the ordinary preparations, and he also objected to their use on account of the smell.

### Papers.

Dr. J. F. Butterfield, of Montrose, read a paper on the subject of "Calculi." His paper was thoroughly discussed by Drs. Helmer, Harger, Rayner, Felton, and others. Dr. Helmer moved that a vote of thanks be given Dr. Butterfield for his valuable paper. The motion was carried.

Owing to the absence of Dr. Philips, his paper on the subject of "Abortion" was read by Dr. Ranck. Dr. Ridge, in discussing this paper, thought that the subject was a very important one. Many farmers are discouraged on account of this trouble in their herds. He advised the use of a subcutaneous injection of a 5 per cent. carbolic-acid solution, 1½ drachms for a dose, and a thorough disinfection of the stables, premises, animals, and isolation of the cows that abort. He finds that with the most rigid treatment he is often unable to check the trouble. The subject was also discussed by Drs. Eves, Benner, Hoskins, Michener, and Pearson. Dr. Pearson thought that the difficulty in treating this trouble is due to the fact that the origin of the disease has not yet been discovered. The infection seems to be carried by the bull. He thought that the carbolic-acid treatment is of very little use, and that the most satisfactory treatment is obtained by the application of local antiseptics to the genito-urinary tract of both bulls and cows. Many cows acquire an immunity to this trouble in two or three years. Nutrition of the animal seems to be an important factor in the progress of this trouble. Dr. Michener said that the cows before aborting show symptoms of uneasiness, and if watched carefully by the owner they can be picked out from the herd. He recommended giving 20 drops of the tincture of aconite every two hours until the symptoms abate. Follow this by 25 drops two or three times a day after the first day. In a few cases they lose the calf. Dr. Ridge differed with Dr. Michener, and said that no premonitory symptoms can be discovered. Dr. Helmer spoke of the difficulty in isolating cases of this kind, and thought our treatment would be much easier if sheds or suitable quarters could be provided. He believed thoroughly in isolation, antiseptics, and disinfection, and believed also that this form of treatment should not be entrusted entirely to the owners, as they are often not competent to carry out the work properly. Dr. McNeil reported cases of abortion in a brood mare which occurred three times in succession. He diagnosed the case as endometritis and treated her with iodine. She was bred afterward and gave birth to a healthy foal. He gave 1½ drachms of the undiluted tincture of iodine once each week for a month.

Secretary Ranck read Dr. Charles W. Boyd's paper on "Rupture of the Flexor Tendons as a Complication of Azoturia." His paper was discussed by Drs. Eves and Michener. Dr. Martein spoke of a similar case which was complicated with osteoporosis. Dr. Carter cited a similar case. Dr. Eves thought it rather doubtful that cases can contract azoturia if they have osteoporosis. He thought their nutrition and condition is not usually such as to predispose to azoturia.

In the paper by Dr. H. P. Eves, of Wilmington—"Peculiar Symptoms Attending Certain Forms of Colic"—he spoke especially of the symptoms noted by the essayist and a few other practitioners, of the peculiar squeaking of the joints in indigestion colic. He said it affects all the joints in the body, and that it resembles the cracking of the joints in the hands of men. Dr. Helmer said that he has observed this condition in horses that are neither sick nor lame. Dr. Eves said that this condition is not noticed in all cases of impaction colic, but in the cases which he has observed it the creaking of the joints ceases when the case recovers from the colic. President Harger suggested to Dr. Eves to study this subject more fully and report to our meeting at some future time.

Dr. James T. Ross spoke of the good results he has obtained in bleeding cows that would not get in calf. He removes six or seven quarts of blood.

Adjourned for lunch at 1.30 P. M.

The meeting was re-convened at 2.30 P. M. Dr. James Mahaffey's paper was the first on the programme. His subject was "Azoturia." His paper was well prepared, and excited an interesting discussion on the subject. Dr. Thomas B. Rayner believes that the treatment of azoturia consists in its prevention. He advises his clients to sit on a fence and read a newspaper as soon as the symptoms of azoturia appear. Dr. Ridge agreed with Dr. Rayner's ideas in reference to resting the animal as soon as the first symptoms are noticed. Dr. Benner said that the man who tries to imitate nature is the most successful, and spoke of the fact that animals in pasture are inclined to rest or stand still rather than to move about after such trouble. He agreed with Drs. Rayner and Ridge. Dr. McNeil noticed that most cases occur in animals which have stood still for two or three days. Dr. Mahaffey asked why it is that this disease occurs oftener in winter than in summer, and thought that the cause must be due to chill. Dr. Eves thought that the reason for this is that horses do not stand still in the summer; if not working they are fighting flies or moving about. Dr. McNeil thought that profuse sweating in the summer also helps to prevent this trouble; also that horses do not drink as much water in winter as in summer. Dr. Benner said that one of the worst cases he ever had was in summer. Dr. Michener thought azoturia is caused by coagulation of the blood, and that exercise is the best treatment. Dr. Weir believed that the trouble in his part of the country during the past winter in this line was due to the feeding of too much corn. Dr. Helmer claimed that there are two varieties of azoturia; the one in the country is much less severe than the form usually seen in the cities. Dr. Fuller thought that food does not play so important a part as exercise, and recommends nitrate of potash in small doses twice a week as a preventive.

"Municipal Milk-inspection," by Dr. J. M. Carter. Dr. Ridge agreed with the essayist from the veterinarian's point of view, but from a producer's stand-point felt that it was entirely too finely drawn, and would recommend that the Association adopt a happy medium in treating this subject. Dr. Mahaffey thought that the essayist exaggerated this subject. Dr. Williams thought that the paper was too close to the facts as they exist, and felt that it would be safe to discuss this subject among ourselves in the same manner as the essayist has treated it; but in discussing it publicly he thought it preferable to keep on neutral grounds. Dr. Michener thought Dr. Carter did not exaggerate the subject. He thought if anything the conditions are worse than he described. He described many disgusting conditions that he had met in his experience, and thought that strong remedies should be applied to overcome the present system of handling milk. Dr. Eves was in favor of making this subject public, so that everybody can know the true facts in the case. Dr. Helmer said that it was difficult to prevent by legislation outbreaks of contagious diseases due to contamination of the milk-supply, but thought that the public should be protected as much as possible from the dangers of handling milk in a careless way. Dr. Butterfield considers the conditions for handling milk are much better than they were a few years ago, but that there is yet much chance for improvement among the ordinary milk dealers and farmers. Dr. Rhoads finds that conditions have improved very much in Chester County in the past few years, but many stables are yet so filthy that it is difficult to treat cases in them without becoming contaminated with stable filth.

The next was a paper by Dr. Pearson on the subject of "Assertions Made by Professor Koch in London at the International Congress on Tuberculosis." A vote of thanks was extended Dr. Pearson for his interesting paper on the subject.

The next business was the adoption of resolutions.

Dr. Pearson moved that Dr. Rhoads be appointed archivist, which motion was put and carried.

Dr. Rhoads made a motion, which was carried, that the selection of a meeting place be left to the Board of Trustees. He also moved that a renewal of the appropriation of $200 for the State Examining Board be granted. This was carried without debate.

Dr. Pearson moved that a rising vote of thanks be extended the outgoing officers for their efficient work.

Next in order was the seating of the new officers, after which the Association adjourned to meet in September.

C. J. MARSHALL,
Recording Secretary.

The following resolutions were adopted at the annual meeting of the Pennsylvania State Veterinary Medical Association, March 4 and 5, 1902:

### NATIONAL HORSE BREEDING COMMISSION.

WHEREAS, It is proposed to erect a National Horsebreeding Commission for the purpose of encouraging the breeding of certain types of useful horses; and

WHEREAS, the good results of the work of this commission will be in proportion to the skill and expert knowledge possessed by its members; be it

*Resolved*, That we heartily approve the principle of the erection of a National Commission to give advice and assistance in respect to the breeding of horses; and be it further

*Resolved*, That we recommend that it shall be provided in the organic law erecting such a commission that the veterinary knowledge and skill of the country shall have membership representation.

### VOTE OF THANKS.

WHEREAS, It was the earnest desire of the members of the State Board of Veterinary Medical Examiners, of this organization, and of the large body of professional veterinarians throughout Pennsylvania, that the work of the Board, its methods, plans, and especially the important work now in progress, should continue along the same successful lines as heretofore; and

WHEREAS, Results depend largely upon practical experience, which it requires time to gain, especially in the office of Secretary, with its immense accumulation of detail and data, beside important daily correspondence; therefore, be it

*Resolved*, That the Pennsylvania State Veterinary Medical Association, in session assembled, extend its approval and a vote of thanks to our respected Governor, Hon. William A. Stone, for his consideration and wisdom shown in the recent reappointment to the office of the Secretary of the State Board of Veterinary Medical Examiners its old and experienced incumbent, Dr. W. Horace Hoskins, of Philadelphia.

### CLINICS AT THE MEETINGS OF THE AMERICAN VETERINARY MEDICAL ASSOCIATION.

WHEREAS, The American Veterinary Medical Association has by its recent policy infringed upon the proper functions of State and local veterinary societies, and to a corresponding degree has neglected its own appropriate field; be it

*Resolved*, That we are emphatically of the opinion that the discussion of questions of purely local and narrow interest is not a proper use of the time of the American Veterinary Medical Association; and especially in view of the fact that many questions of general and broad interest have to remain undiscussed, owing to the crowded condition of the programme; be it further

*Resolved*, That we hereby protest against the continuance of the practice of holding surgical clinics under the auspices of and in conjunction with the meetings of the American Veterinary Medical Association. We deem such exhibitions of no educational value, but are calculated to obscure the proper function of the Association, and are injurious to the profession in the locality in which they are held.

### DR. RUSH SHIPPEN HUIDEKOPER.

WHEREAS, We have lost through death our beloved friend and colleague, Dr. Rush Shippen Huidekoper, one of the greatest builders of the veterinary profession in America; be it

*Resolved*, That with a sense of profound loss and sorrow, and with the wish to record in permanent form our appreciation of the life and services of Dr. Huidekoper, a brief history of his career shall be prepared by the Resolution Committee and spread upon the minutes of this Association; and be it further

*Resolved*, That our sincere sympathy is extended to Mrs. Huidekoper in her great affliction.

### MRS. D. E. SALMON.

WHEREAS, Through the death of Mrs. Salmon, Dr. D. E. Salmon has sustained the loss of a loving and helpful consort, and society has been deprived of a gentle, refining influence; be it

*Resolved*, That we offer to Dr. Salmon our deepest sympathy in his loss and bereavement.

DR. W. HORACE HOSKINS.

WHEREAS, Our esteemed colleague, Dr. W. Horace Hoskins, has been reappointed a member of the State Board of Veterinary Medical Examiners by the Governor; be it

*Resolved*, That we, the members of the Keystone Veterinary Medical Association, in regular meeting assembled, hereby express our unqualified approval of this appointment, and tender the Governor, Hon. William A. Stone, our thanks as an organization and as individuals for this well-merited recognition of Dr. Hoskins' strenuous and self-sacrificing services for the veterinary profession and for the Commonwealth as an active member of the State Veterinary Examining Board; be it further

*Resolved*, That this resolution be spread on the minutes of this Association and that a copy be forwarded to his Excellency, the Governor.

Done at Philadelphia, this eleventh day of February, 1902.

Attest:

WARREN L. RHOADS,
President.

E. M. RANCK,
Secretary.

WHEREAS, There has already been much valuable work done at the University of Pennsylvania and is now being done under the direction of our esteemed colleagues, Dr. Leonard Pearson and Dr. M. P. Ravenel, in the advancement of the study of tuberculosis and tuberculin; and

WHEREAS, This work has not only been most thorough, but the reports upon said work most thoroughly systematized; be it

*Resolved*, That we hereby encourage the continuance of this work by showing our professional appreciation at this time by a unanimous vote of commendation.

WHEREAS, The splendid work of our Pennsylvania State Live-stock Sanitary Board continues to attract attention and recognition at home and abroad; and

WHEREAS, Its generous support and approval by the people of our own State is a source of much gratification to this Association; therefore, be it

*Resolved*, That these most excellent results have largely followed the continuance in place and power of our colleague, Dr. Leonard Pearson; be it further

*Resolved*, That we most highly commend the action of our Governor in retaining our colleague in office in this Board, and our Legislature in continuing its financial support to the work of the Board, thus expressing confidence and appreciation of the successful work already accomplished.

THANKS TO THE H. K. MULFORD COMPANY.

WHEREAS, We have been instructed and entertained by a visit to the extensive and finely equipped and carefully conducted laboratories of the H. K. Mulford Company, and have dined as guests of this company; be it

*Resolved*, That our thanks are hereby tendered to this firm, and we assure them that the visit was very much enjoyed, and their hospitality is appreciated.

WHEREAS, The American Veterinary Medical Association at its annual convention at Atlantic City, in Septemer, 1901, placed itself on record as to the ungrateful, unprofessional, and cowardly actions of Dr. Claude D. Morris, and justly visited upon him the condign punishment of summary expulsion from its roll of membership; and

WHEREAS, This Association, in convention assembled, approves of this prompt and proper action of that Association; be it further

*Resolved*, That this Association, in commending this action, equally regrets and regards with great concern the attitude of the New York State Veterinary Society in continuing to condone this the most flagrant act of treachery in the history of veterinary medicine in America.

### UNITY PLEDGE.

WHEREAS, The State of Pennsylvania has developed a code of veterinary laws which, in governing the practice of veterinary medicine and the control of the diseases of animals, are the best in the country, but are still imperfect, and as knowledge grows and new conditions develop will require alterations; and

WHEREAS, Such legislation as now exists was secured through the efforts of the veterinary profession of the State acting as a unit, all difficulties being settled in convention, and a united front being presented to the Legislature; and

WHEREAS, Much of the failure to secure equally good conditions in other States may be traced to dissensions in the profession itself; be it

*Resolved*, That we, the members of the Pennsylvania State Veterinary Medical Association, in convention assembled, realizing the advantages resulting from a policy of unity and the suicidal folly of dividing and urging conflicting measures and recommending opposing candidates, hereby pledge ourselves and our Association, that in all matters of interest to us as a profession to strive as a harmonious and united body for the purposes decided to be for the best interests of this body.

### MICHIGAN STATE VETERINARY MEDICAL ASSOCIATION.

I HEREWITH transmit a short summary of the proceedings of the last meeting held by our Association, at Lansing, February 4th and 5th, 1902. The report is necessarily short; in fact, it is merely a synopsis of actual work done, without comment.

Meeting called to order February 4th, by President J. J. Joy. Forty members answered the roll-call.

President's annual address by Dr. Joy.

Committee appointed to arrange programme of entertainment for the ladies.

Minutes of previous meeting read.

Eight new members were elected.

Letters from absent members read.

W. W. Thorburn, Secretary of State Veterinary Board, was expelled.

Dr. J. Hawkins, Prof. Charles E. Marshall, of the M. A. C., and Dr. H. P. Baker, Secretary of the State Board of Health, were elected honorary members of the Association.

Resolutions of sympathy on the deaths of Mrs. Wooley and Dr. R. E. Hunt were adopted.

Delinquent members suspended for non-payment of dues.

The following officers were elected, viz.: President, H. F. Palmer, Detroit; First Vice-President, H. M. Gohn, St. Johns; Second Vice-President, J. Harrison, Maple Rapids; Third Vice-President, H. S. Smith, Albion; Secretary and Treasurer, W. A. Giffen, Detroit; Directors —Judson Black, J. W. Brodie, D. G. Sutherland, William Jopling, J. J. Walkington, and A. McKercher.

Secretary's report read.

A resolution was adopted giving the Secretary $15 in addition to his salary for the year's work.

Treasurer's report was read, showing a balance of $130.16.

Committee on Diseases reported.

State live-stock sanitary laws freely discussed.

"*Resolved*, That the Committee on Legislation be empowered to draft amendments to the State live-stock sanitary laws, and that said committee be instructed to take such action as may be deemed best to have the amendments brought before the Legislature at its next session.

Committee on Intelligence and Education reported.

The banquet was held on the evening of February 4th.

Dr. George W. Dunphy acted as toastmaster. Toasts: "Our Association, Drs. Brenton, Sutherland, Whitney, Clement, Byers, and Cummings; "Legislation," Dr. F. C. Wells; "Our New Members," Dr. H. L. Bellinger; "Our Officers," Dr. H. M. Gohn; "Our Guests," Major H. E. Johnson and Dr. Davis; "Our Bachelor Members," Drs. Smith and McKercher; "Our State Board," Dr. H. F. Palmer; "The Ladies," Drs. Giffen and Jopling.

February 5th. Dr. Brenton, assisted by Drs. Campbell, Joy, Sutherland, Black, and others, demonstrated the following surgical operations, viz., neurectomy for the cure of lameness, shaking the head involuntarily, and cribbing; tenotomy for the relief of stringhalt and bone spavin; operations for spaying in the mare and for the removal of lateral cartilages were performed.

The following papers were read and discussed, viz.: "Contagious Diseases of Live-stock in Michigan," Dr F. C. Wells; "The Work of the Michigan Agricultural College," Dr. Smith; "The Relation of the Michigan Agricultural College to Veterinary Science," Dr. G. A. Waterman; "Veterinary Protection," Dr. Charles Nyce; "Diagnostic Agents," Dr. H. F. Palmer; "The Horse's Stomach," Dr. William Jopling; "Azoturea," Dr. J. J. Walkington; "Anthrax from the Bacteriological Standpoint," Dr. C. E. Marshall.

The following resolutions were carried unanimously, viz.:

"*Resolved*, That the thanks of the Association be extended to Hon. A. T. Bliss, Governor, for the kind consideration and many courtesies he has shown us during his term of office.

"*Resolved*, That the Association's thanks be extended to Hon. J. E. Weter for the cheerful and valuable assistance he has given us in our legislative work.

"*Resolved*, That the Association put itself on record as favoring higher veterinary education in colleges and a curriculum of at least three years."

Dr. Joy installed the newly elected President, Dr. Palmer.

The President appointed the following committees: Legislation—Drs. Giffen, Wells, Dunphy, Black; Intelligence and Education—Drs. Whitney, Smith, Waldron; Diseases—Drs. Wells, Gohn, Bellinger; Clinics—Drs. Waterman, Manning, Irwin; Finance—Drs. Hamilton, Farmer, Muir.

Motion to adjourn carried.

W. A. GIFFEN,
Secretary.

### MASSACHUSETTS VETERINARY ASSOCIATION.

THE eighteenth annual meeting of the Association was held at the Hotel Cecil, Wednesday evening, April 23d, at 6 o'clock. The members present were Drs. Blackwood, Beckett, Bunker, Emerson, Harrington, Howard, May, Peters, Pierce, Rogers, Winchester.

Applications for membership were received from Dr. John T. Conners, of South Boston, and Dr. George T. Quinlan, of Brookline. Under the By-laws both names were laid over until the next meeting for action.

The election of officers for the ensuing year resulted as follows: President, Dr. B. D. Pierce; First Vice-President, Dr. H. P. Rogers; Second Vice-President, Dr. George Lee; Secretary and Treasurer, Dr. Edward T. Harrington; Executive Committee—Drs. Austin Peters, Thomas Blackwood, J. R. McLaughlin, W. L. La Baw, M. Bunker.

There was a general discussion as to the advisability of changing our place of meeting as a means of increasing the attendance.

It was voted to refer to the May meeting for definite action the following motion:

"*Resolved*, That the contract for the ensuing year with the Boston Medical Library be not renewed, and a contract be entered into with the Boston Veterinary Hospital to the same end if satisfactory arrangements can be made."

The resignation of Dr. Etienne, of St. Hyacinthe, P. Q., was read and accepted, and it was voted that the Secretary send him a letter of regret.

After the business meeting the members sat down to the banquet and passed a very enjoyable evening.

After the banquet the following toasts were proposed by the toastmaster, Dr. L. H. Howard: "Our Association," responded to by Dr. D. B. Pierce; "One of Our Oldest Members Honored by the National Association," Dr. J. F. Winchester; "Harvard Veterinary School," Dr. E. C. Beckett; "The American Veterinary College," Dr. M. Bunker; "The Commonwealth of Massachusetts," Dr. Austin Peters; "McGill University," Dr. T. Blackwood; "Our Out-of-town Members," Dr. D. Emerson; "The Bureau of Animal Industry and Sanitary Police," Dr. H. P. Rogers;

"Our Secretary," Dr. E. T. Harrington; "The Profession Across the Water," Dr. H. S. Lewis; "The Ladies," Dr. A. W. May.

The meeting then adjourned.

EDWARD T. HARRINGTON, V.S.,
Secretary.

### NEW JERSEY BOARD OF VETERINARY MEDICAL EXAMINERS.

THE New Jersey Board of Veterinary Medical Examiners met at the State House on Monday, May 5th, and before noon of that day the oath of office was administered by Assistant Secretary of State J. B. R. Smith. Governor Murphy's selection to comprise this board consists of these names: Dr. William Herbert Lowe, Paterson; Dr. J. E. Smith, Jersey City; Dr. Whitfield Gray, Newton; Dr. T. Earle Budd, Orange, and Dr. T. B. Rogers, Woodbury. Immediately after the oath was taken an organization was completed and the following officers elected: President, Dr. William Herbert Lowe; Secretary, Dr. Whitfield Gray, Newton; Treasurer, Dr. T. E. Smith.

A committee was named consisting of the President, Secretary, and Dr. Thomas B. Rogers to arrange the details of the approaching examination.

It was decided to hold the first examination at the State House, Trenton, on June 24, 1902, and intending applicants can secure the desired information and application blanks from the Secretary.

Immediately after the organization and the necessary business completed the meeting adjourned as a mark of respect to the father of the "McKee act," Senator Wood McKee, of Paterson, whose father's death occurred the day previous.

It is perhaps needless to add that this was an eventful day to the veterinary profession of the State, because it really marks the beginning of a new and important period in its history, and places the members of the profession on a plane with other branches of medicine. A careful perusal of the McKee act, which is responsible for the existence of this commission, will show that hereafter in New Jersey intending practitioners must be properly educated and in other ways fitly qualified.

A united feeling exists among the profession in the State, and this harmonious condition, together with the recent legislative recognition, is due largely to the untiring efforts of the President, Dr. William Herbert Lowe.

WHITFIELD GRAY,
Secretary.

